# On the Probable Sensations of the Head, after Being Severed from the Body

**Published:** 1799-03

**Authors:** 


### *On the probable Senfations of the Head, after being fevered from the Body.*

AMONG other fingular queftions lately agitated in France and Germany, the following is not the leaft curious: "*Whether the feparated head of a perfon fuffering on the fcaffold be ftill, for a certain time, confcious of its fufferings?*" This queftion was propofed at the time, when the guillotine was firft adopted as the inftrument of public execution in France; and the writings which appeared on this apparently whimfical fubject, were numerous. Prof. SOEMMERING maintained in a particular treatife, firft printed in the *Moniteur*, that the confcioufnefs of the fufferer ftill continued for fome time; and the Profeffor is fo far perfuaded of the truth of this affertion, that, in his opinion, the fevered head would ftill emit founds, if it were not feparated from the organs of fpeech, as well as thofe of refpiration.

Prof. WEDEKIND controverted this opinion, and defended the French method of beheading as the quickeft and leaft painful fpecies of death, by which all confcioufnefs is at once intercepted and annihilated: he fays, that the blood-veffels of the feparated head are *ipfo facto* inftantly and completely emptied of the blood, and that the organ of the mind is thus rendered totally inactive; confequently the head cannot be confcious, either of its former or prefent ftate.

Mr. CLOSSIUS, of Tubingen, though not fully convinced that the head, in a differered ftate retains confcioufnefs, yet confiders this fact as highly probable, and on the whole, agrees in opinion with Soemmering. He dwells at confiderable length, in his treatife "*On Decapitation*" upon the objections ftarted by Wedekind, and endeavours to prove that the blood-veffels of the head are by no means fo quickly and completely emptied, as the Profeffor is difpofed to think; and that even without an actual circulation of the blood, there may ftill remain fome activity and energy in the brain. He produces in fupport of his hypothefis, a variety of arguments, derived partly partly from analogy and a comparifon of the fymptoms of a perfon beheaded with thofe obferved in fimilar fituations; and partly from appearances which have been noticed in individuals executed by the fword or guillotine. On account of this probability, ought not the dictates of humanity to be liftened to, and inftead of beheading, fhould not another mode of execution be devifed, by which the deadly blow, and the complete annihilation of the natural ftructure of the brain, might take place at the fame inftant? Such, for inftance, would be a fudden and violent convulfion of the brain, a mortal thruft on the ftomach, and the like.

Another German writer on this fubject, C. A. Eschenmayer, combats the opinion of Soemmering, but deals fo much in confufed philofophic fpeculations and vague hypothefes, that we fhall forbear taking notice of what he advances; and proceed to give fome account of a French treatife entitled, "*Opinion du Citoyen* Sue, *fur le Supplice de la Guillotine*," a Paris, 16. pp. 8vo.—Citizen Sue, not contented with afferting, that the head ftill continues to live, and to feel the moft excrutiating pain, as long as the blood remains warm in the fmalleft veffels, maintains that the lower part of the body alfo muft fuffer, after the head has been ftruck off. His arguments are not a little curious. He compares the act of beheading with that of amputation of a limb; as it has been obferved that perfons have felt, or at leaft imagined that they felt pain in a limb, after being feparated from the body by a chirurgical operation, as alfo after a diflocation of the vertebræ; that the upper parts of the body remain fenfible and alive, while the lower extremities are lifelefs and infenfible to pain. The incongruity of this mode of reafoning from analogy is fo palpable that it requires no refutation.

The author quotes various inftances of preternatural births, or monfters born without a brain, and fpinal marrow, on which he grounds his conjecture, that the lower extremities of the body are ftill fenfible to pain, after a feparation of the head has taken place. But, befides the obvious inconfiftency in this comparifon, it requires to be previoufly proved, that fuch monfters have ever had confcioufnefs, and fuffered pain, on account of the unnatural ftructure of their bodies. The remarkable facts witneffed by the author, during a number of executions at Paris, and related in this treatife; for inftance, the fingular phenomenon of fmiling, or rather of blufhing, in the trunklefs head of Madame Cordet, the affaffin of Marat, are no doubt curious, but not conclufive.

The fubject of difpute is indeed fuch as admits of no pofitive decifion; for the feelings of a perfon, after the mortal blow has been given by the fword or guillotine, cannot be afcertained, and we may pronounce it impoffible sible for human reason to form an adequate conception of their greater or less duration. If there exist a something within the body, which after its dissolution hastens towards another destination, in which we shall not be insensible to agreeable or disagreeable sensations, this something will no doubt also possess the consciousness of its situation, whether the body has been destroyed by a putrid fever, by poison, by a concussion of the brain, or by any other mode of depriving or taking away life. It does indeed not, here, depend upon the state of the circulation of the blood, or other relations, as connected with the body; for it must be admitted, that this something, if it continue after and without the body, has no connection with corporeal causes, organs of the mind, or the like. W.

